# Supplementary material for: A methodology for the design of experiments in computational intelligence with multiple regression models
Source: PeerJ. 2016 Dec 1;4:e2721. doi: 10.7717/peerj.2721 (PMC5136129; doi:10.7717/peerj.2721)
Supplement: Data S1 — Datailed results from UC Irvine Machine Learning Repository (Housing, Machine CPU, Wine Quality, Automobile and Parkinson) and the 3 Use Cases (Protein Corona, Gajewicz Metal Oxides and Aquatic Toxicity) [file peerj-04-2721-s001.zip › 6_UseCases/2_GajewiczMetalOxides/NN.details.csv.repeatedcv.split3.pdf]

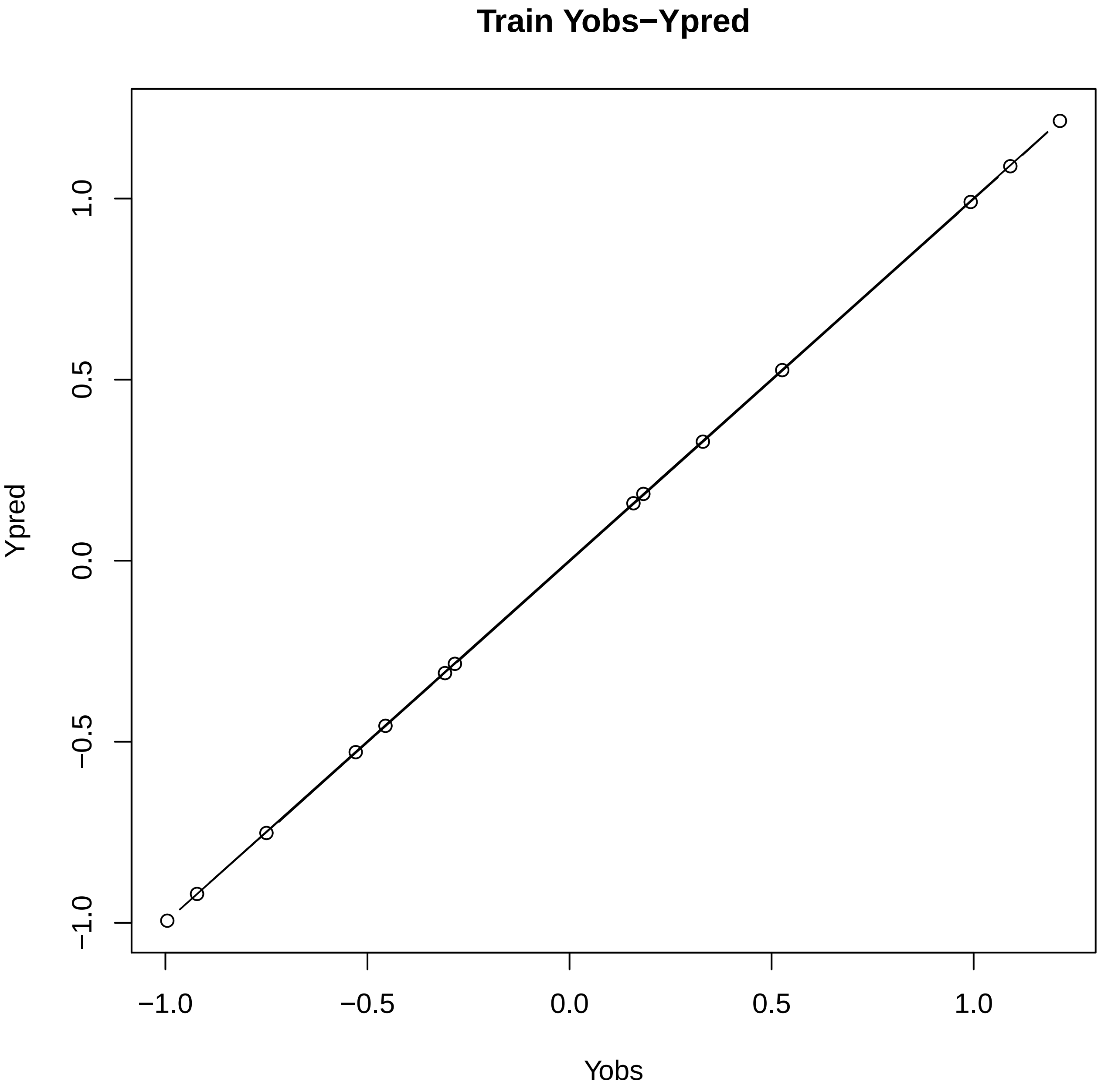
Train Yobs−Ypred
Ypred
1.0
0.5
0.0
−0.5
−1.0
Yobs
−1.0
−0.5
0.0
0.5
1.0

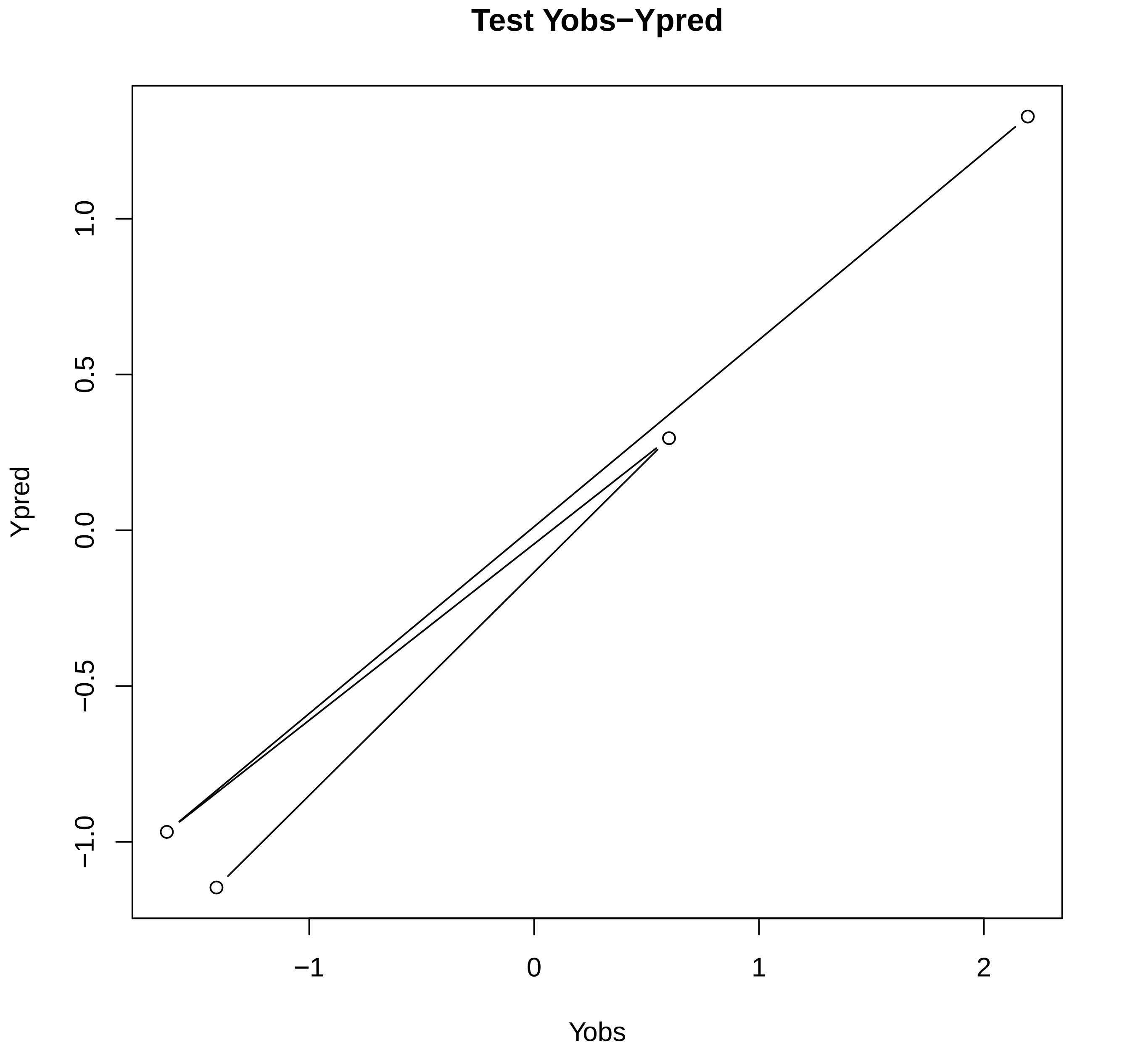
Test Yobs−Ypred
Ypred
1.0
0.5
0.0
−0.5
−1.0
−1
0
1
2
Yobs

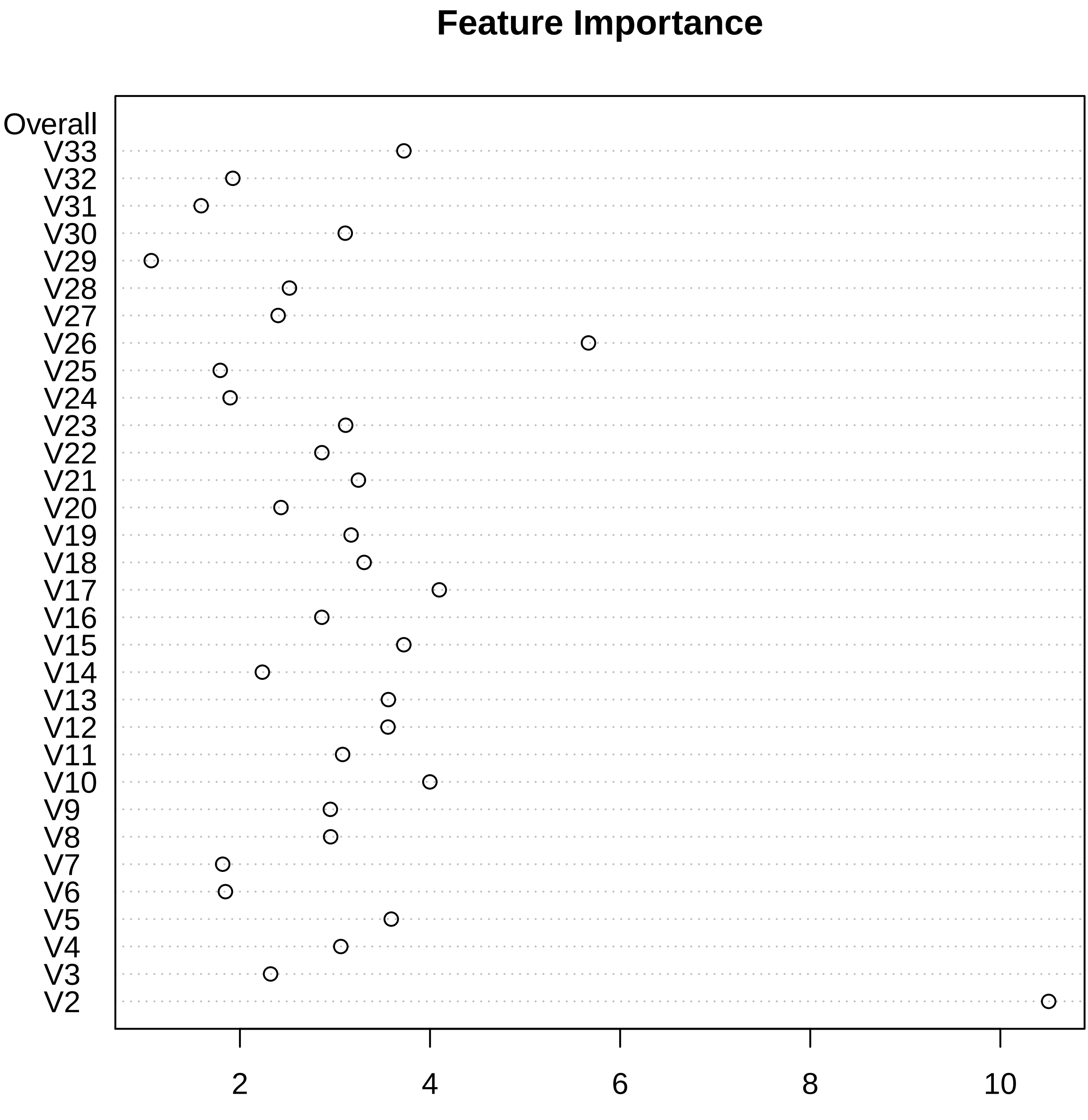
Feature Importance
Overall
V33
V32
V31
V30
V29
V28
V27
V26
V25
V24
V23
V22
V21
V20
V19
V18
V17
V16
V15
V14
V13
V12
V11
V10
V9
V8
V7
V6
V5
V4
V3
V2
2
4
6
8
10

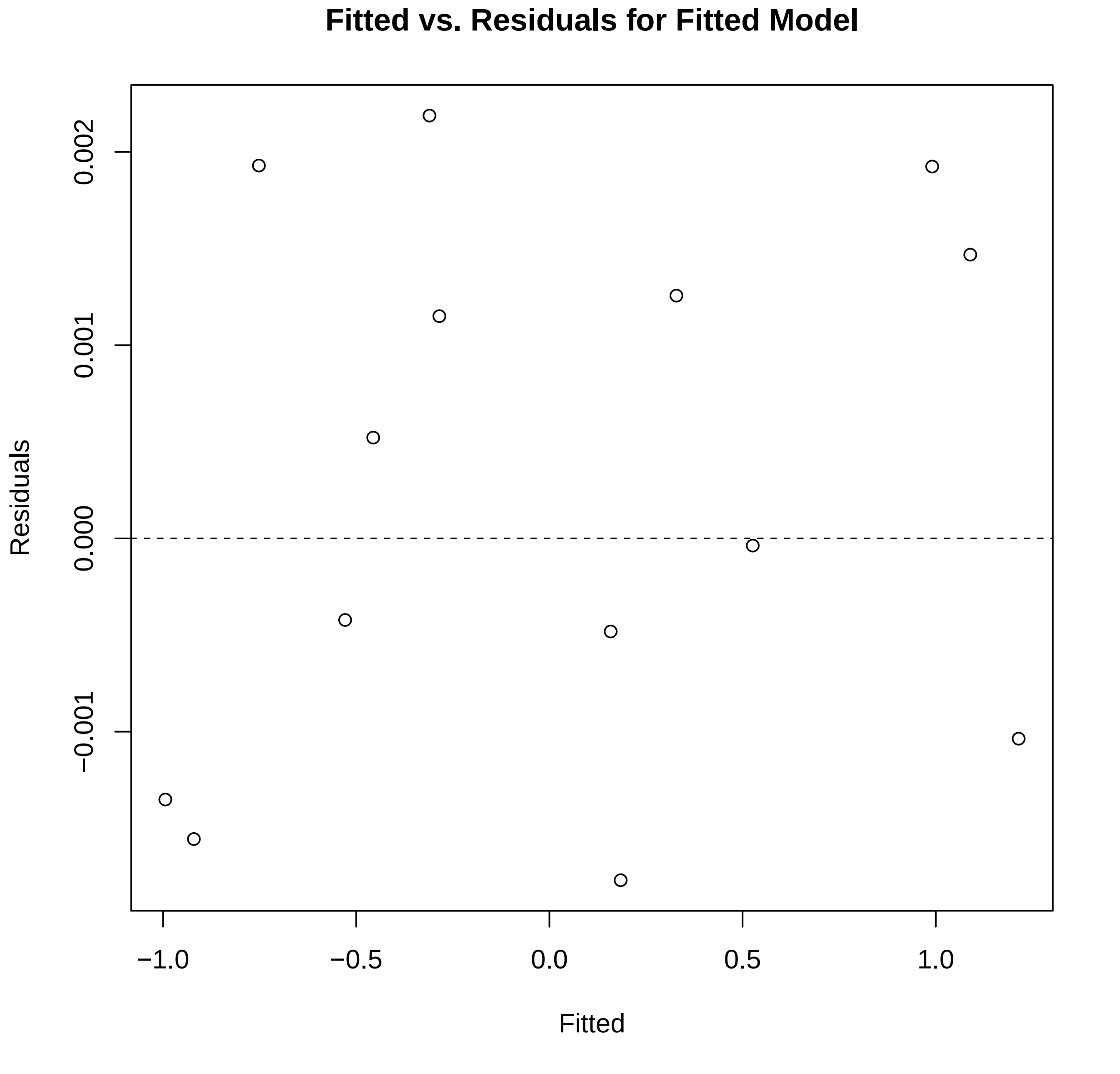

Fitted vs. Residuals for Fitted Model
Residuals
0.002
0.001
0.000
−0.001
−1.0
−0.5
0.0
0.5
1.0
Fitted
